# Application of a Cutting Guide to Optimize Fit of 3D-Printed Patient-Specific Implants in Reconstruction Following Resection of Osseo-Invasive Tumors

**DOI:** 10.7759/cureus.102281

**Published:** 2026-01-25

**Authors:** Srujana Venkata Vedicherla, Zakir Chew, Ming Li Chia, Adriel Leong, Yan Lin Yap, Vincent Diong Weng Nga

**Affiliations:** 1 Neurosurgery, National University Hospital, Singapore, SGP; 2 Plastic Surgery, National University Hospital, Singapore, SGP

**Keywords:** cranioplasty, decompressive craniectomy, osseo-invasive tumours, patient specific implant, peek: polyetheretherketone

## Abstract

In managing osseo-invasive tumors, such as meningiomas, the treatment of choice is complete resection, including the dural origin and any involved bone. Virtual preoperative surgical planning, combined with tailored intraoperative reconstruction, can optimize outcomes. Repairing bony defects with patient-specific implants (PSIs) in a single-stage procedure has been shown to reduce operative time and morbidity. Here, we present the use of a resection template to further optimize reconstruction outcomes. We analyzed five patients with cranial tumors exhibiting significant bony invasion who underwent surgery at our institution between June 2023 and January 2025. Tumor resection was virtually planned in collaboration with PSI manufacturers (Synthes^®^, West Chester, PA, USA, and Protomed^®^, Westminster, CO, USA), who produced the PSI and corresponding cutting guide for intraoperative use. Among five patients (four men and one woman; mean age: 60 ± 13 years), the mean tumor size was 54.3 ± 12.2 mm × 55 ± 16.4 mm, and the mean operative time was 3.6 ± 1.6 hours. In all cases, resection proceeded as planned, with accurate PSI placement and satisfactory postoperative fit confirmed via magnetic resonance imaging. This retrospective five-case institutional series demonstrates the feasibility of single-stage resection and reconstruction of osseo-invasive cranial tumors using PSI and a resection template to improve fit and reduce intraoperative handling and lower infection risk. This may offer excellent oncological, functional, and cosmetic outcomes by enhancing implant fit and provides simple, accurate, and efficient surgical solution.

## Introduction

Osseo-invasive cranial tumors-including meningiomas, solitary fibrous tumors, and metastatic lesions-require complete resection of the tumor and the affected bone and dura (e.g., Simpson grade I resection for meningiomas) to achieve optimal long-term outcomes. Meningiomas are the most common primary brain tumors, accounting for approximately 34% of all central nervous system tumors. They most frequently occur at the convexity and parasagittal regions and may directly invade the bone or cause secondary hyperostosis. Intraosseous meningiomas are rare, with primitive/primary intraosseous meningiomas (PIMs) being even less common. PIMs typically originate within the fronto-sphenoidal bone and may secondarily involve the dural sheet. In contrast, plaque or nodular meningiomas arise from the arachnoid and may invade the bone [1]. Although rare, comprising only 1%-2% of all meningiomas, PIM accounts for approximately 67% of extradural meningiomas [2,3]. Metastatic tumors (e.g., those from the breast, lung, or prostate) may also invade the dura and adjacent bone.

The complexity of surgically managing these lesions is complicated by their irregular size and deep, unpredictable bone invasion, making high-quality reconstruction difficult. Delayed cranioplasty, involving initial resection of osseo-invasive tumors and followed by patient-specific, fabricated, customized cranial implants, has been performed but requires additional surgery, general anesthesia to repair the skull defect, and prolonged exposure to skull deformity [4]. Single-stage cranioplasty using hand-molded titanium mesh or polymethylmethacrylate (PMMA) carries higher postoperative infection rates and poorer cosmetic outcomes.

Historically, different materials have been used for cranioplasty, including PMMA, titanium, porous polyethylene, and hydroxyapatite [5]. Polyetheretherketone (PEEK) implants are widely regarded as the preferred choice for cranioplasty [6], craniofacial defect repair [7], and skeletal reconstruction [8,9], offering radiolucency, chemical inertness, durability, and comfort; they also avoid imaging artifacts and do not conduct heat [6,10].

Advances in computer-assisted manufacturing and three-dimensional (3D) printing techniques have enabled single-step surgeries using patient-specific implants (PSIs) designed from preoperative 3D CT scans [7,11-14]. Therefore, virtual preoperative surgical planning with tailored reconstruction after tumor resection can optimize outcomes. Repairing bony defects with PSI in a single-stage procedure reduces operative time and morbidity [15,16]. Despite using PSI, restoring skull contours for optimal function and cosmetic outcomes remains challenging due to suboptimal fit, bone loss, and difficulty with skin closure. Therefore, this study aims to report the use of a commercially available resection template to optimize PSI fit in a single-step procedure, to improve resection and reconstruction outcomes, and present our institutional experience with a case series of five patients.

## Case presentation

Patient recruitment and surgical details

We included five patients with osseo-invasive tumors who underwent surgery at our institution from June 2023 to January 2025. The resection strategy was preplanned and shared with the manufacturer (Synthes^®^, West Chester, PA, USA, and Protomed^®^, Westminster, CO, USA, PSI), who provided the PSI and cutting template used for reconstruction.

Demographic, clinical, and radiological data were collected. All five patients (four men and one woman) had osseo-invasive cranial tumors. The mean tumor size was 54.3 ± 12.2 mm × 55 ± 16.4 mm with a mean surgical duration of 3.6 ± 1.6 hours. Tumor types included four meningiomas (WHO grades I-III) and one solitary fibrous tumor (WHO grade III). Four cases were primary resections, and one was a recurrence. Simpson I resection was achieved in one case, and Simpson II in the others. PSI fit was excellent in all cases, confirmed via postoperative magnetic resonance imaging (MRI). No perioperative infections occurred. Cosmetic and functional outcomes, evaluated using the Sloan classification, were Class 1 or 2 in all patients [17]. In all cases, we used the custom-made PEEK prostheses, with stiffness and strength similar to bone (Synthes^®^ and Protomed^®^ PSI).

Preoperative virtual planning and manufacture of the resection frame and PSI

**Figure 1:**
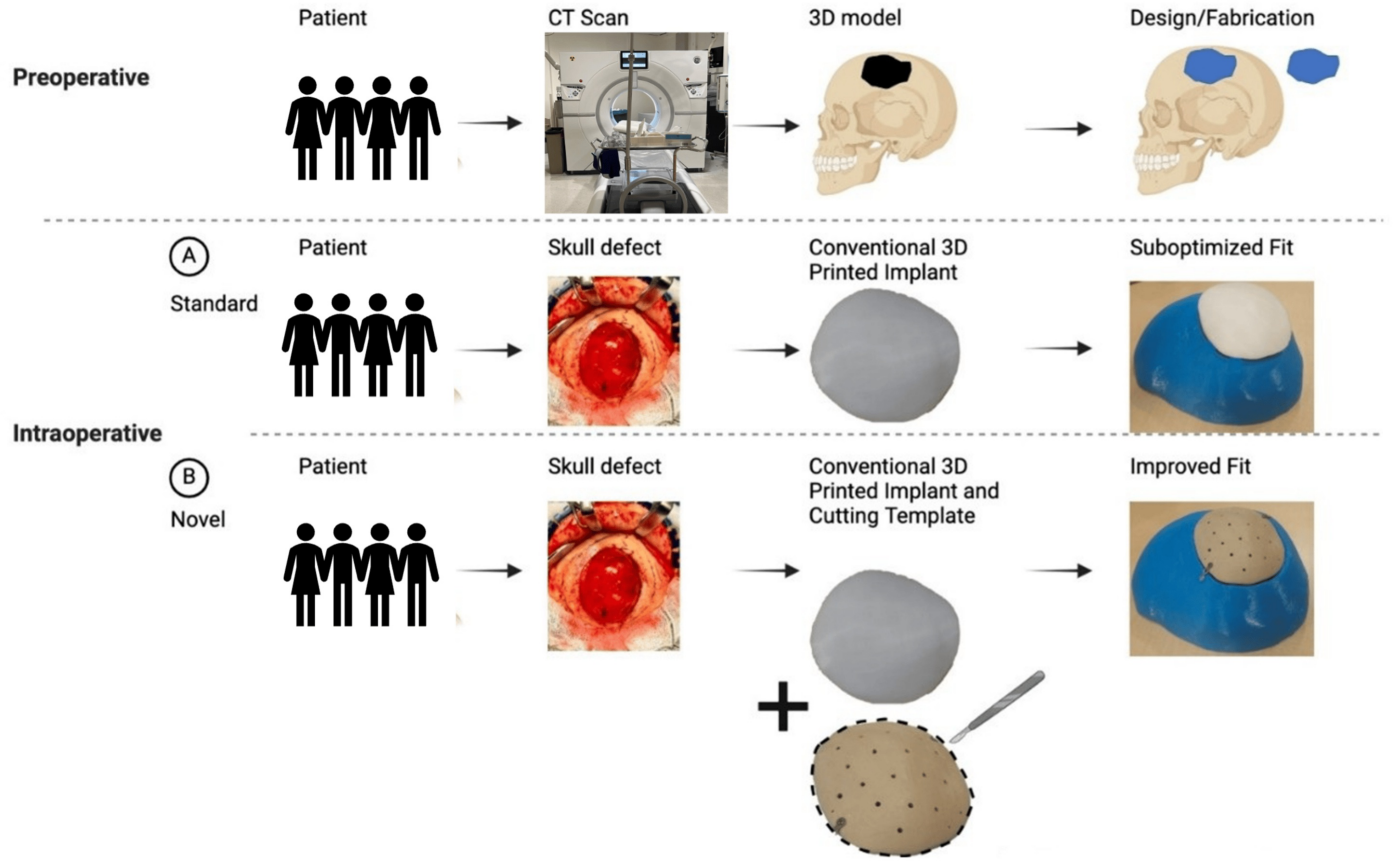
Preoperative virtual planning and 3D printing of patient-specific implant. Standard application of implant and use with an intraoperative cutting template shown to better optimize implant fit 3D: three-dimensional The figure was created by the authors using Microsoft PowerPoint.

Preoperative fine-cut CT scans were performed for each patient using preset parameters to optimize reconstruction: 512 × 512 matrix, 1 mm slice thickness, 1 mm feed per rotation, 1 mm reconstructed slice increment, reconstructed algorithm bone, and 0° gantry tilt. CT datasets were downloaded and sent in Digital Imaging and Communications in Medicine format to the manufacturer (Synthes^®^ and Protomed^®^ PSI). A brain MRI was also performed to better define the relationship of the tumor to the brain parenchyma. The surgeon preplanned the tumor resection strategy and discussed it via web conference with the engineer from the manufacturing team. Virtual resection was planned on a 3D model, with bony cuts mapped on CT slices (TruMatch, Warsaw, Indiana, USA, Comprehensive Solutions in Facial Reconstruction). The bone was considered pathological if it appeared thicker, with no distinction between the cortical and spongiosa layers. A supplementary safety margin of at least 10 mm was applied, except in areas near the frontal sinus (except in one case of its major involvement) and the superior sagittal sinus (SSS). After the resection simulation was approved, the patient-specific prostheses were designed to cover the bone defect using computer-aided design software. Furthermore, a surgical guide was designed as a frame complementary to the prosthesis (Figure 1).

Operative technique with postoperative analysis

All patients were administered preoperative antibiotics within three minutes of skin incision. The incision and surgical approach to the tumor were preplanned using StealthStation™ (Medtronic, Dublin, Ireland) navigation to guide resection and reconstruction. After raising the skin flap, the planned resection template was placed on the bone, and the resection margin was marked. Final validation was performed using navigation tools before craniectomy and resection. Craniectomy was extended if diseased bone was observed on direct visual inspection within a 10 mm margin to allow implant placement. Subsequently, standard tumor resection and debulking were performed. The cranioplasty implant was placed in an onlay design and secured with titanium screws. Patients were administered three doses of intravenous antibiotics postoperatively, with routine subgaleal drains placed for 24 hours postoperatively before removal. Postoperative brain MRI was performed to assess the extent of tumor resection and cranioplasty fit. Wounds were reviewed 10-14 days postoperatively for suture or staple removal in the clinic.

Clinical cases

Between June 2023 and January 2025, a total of five patients with osseo-invasive tumors presented to our institution and were thus recruited for this study. All five patients were non-diabetics and not immunocompromised. Radiotherapy and chemotherapy, if required, were initiated after adequate wound healing was confirmed on clinical review.

Case 1

**Figure 2:**
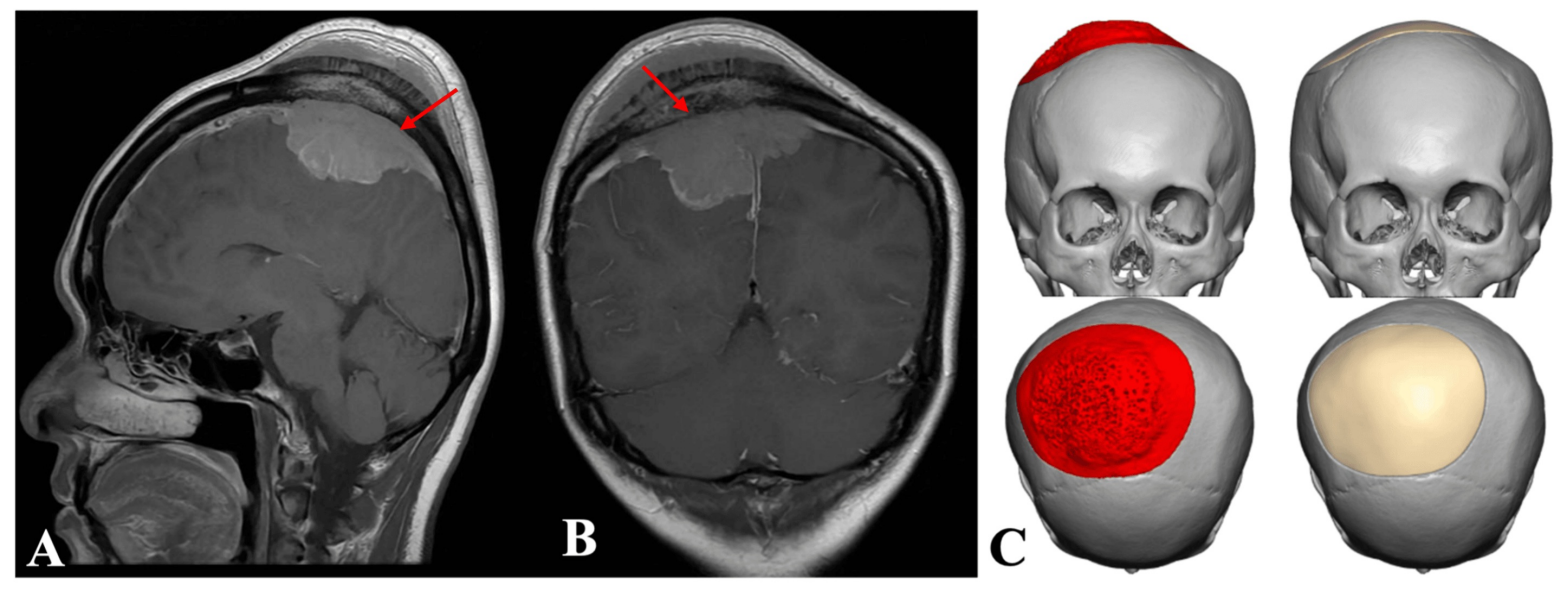
(A) Sagittal and (B) coronal preoperative images of MRI brain (T1-weighted with contrast) showing right parasagittal tumor (red arrow). (C) Preoperative implant design MRI: magnetic resonance imaging

**Figure 3:**
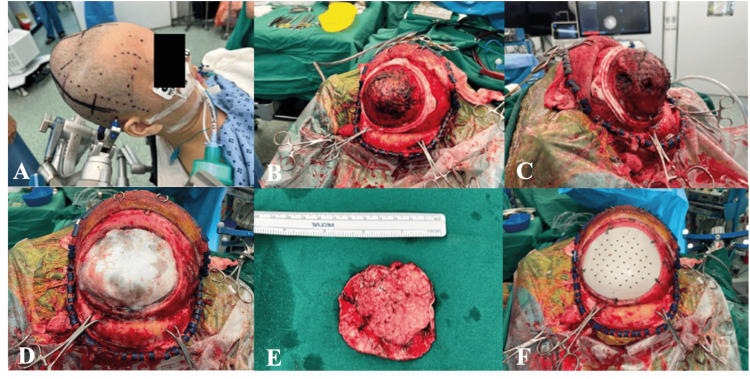
(A) Surgical positioning and markings prior to resection. (B, C) Cutting template placed preresection. (D, E) Post-craniectomy with tumor resected followed by (F) PEEK implant fixation PEEK: polyetheretherketone

A 45-year-old woman presented with left-sided weakness, numbness, and a progressively enlarging scalp lump for a few months. Brain MRI revealed a right frontal parasagittal tumor with calvarial invasion: a lobulated, enhancing extra-axial mass measuring 6.4 × 6.9 × 3.2 cm centered at the right paracentral vertex, with extensive surrounding dural and falcine thickening and enhancement. Simpson II surgical resection was achieved via a bicoronal approach. Histology revealed a WHO grade I meningioma. Figures 2, 3 illustrate preoperative and intraoperative brain MRI images. Postoperatively, she experienced seizures that were controlled with anticonvulsants. Small remnants over the anterior and posterior SSS increased in size on follow-up and were treated with Gamma Knife Surgery (GKS) 11 months post-surgery (three different lesions measuring 5.7, 1.7, and 0.5 cc). All lesions were treated with a prescription dose of 13 Gray (Gy).

Case 2

**Figure 4:**
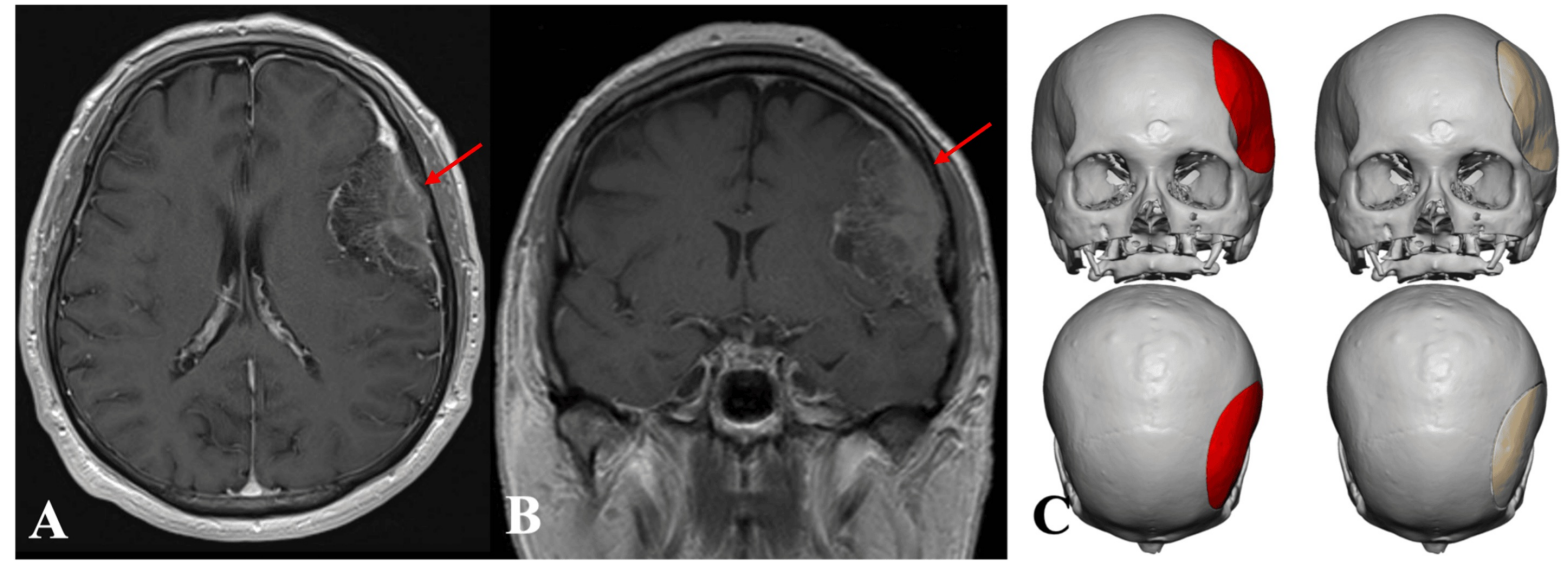
(A) Axial and (B) coronal preoperative images of MRI brain (T1-weighted with contrast) showing left convexity tumor (red arrow). (C) Preoperative PEEK implant design images MRI: magnetic resonance imaging; PEEK: polyetheretherketone

**Figure 5:**
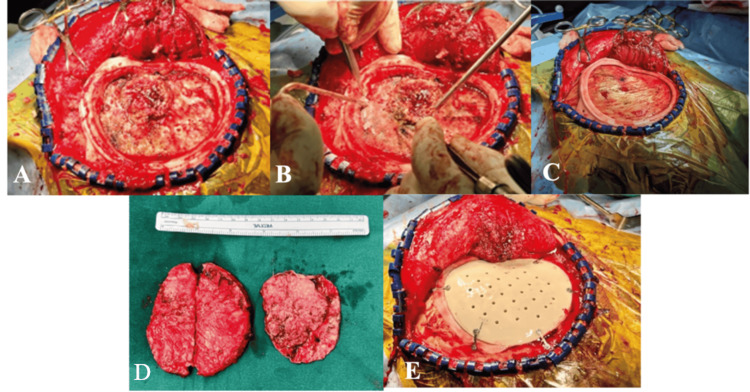
(A, B) Intraoperative images with (C) cutting template positioned and (D) tumor resected followed by (E) PEEK implant fixation PEEK: polyetheretherketone

A 70-year-old man presented with diplopia and a right homonymous superior quadrantanopia. He had a history of hypertension, hyperlipidemia, ischemic heart disease on aspirin, right renal cell carcinoma, and a thymic mediastinal mass lesion. Brain MRI revealed an enlarging left frontal convexity tumor with calvarial invasion: a large enhancing extra-axial mass measuring 3.7 × 5.7 cm eroding the inner table of the adjacent left frontal bone, with abnormal hypointense marrow signal but sparing the outer table of the skull (Figure 4). Simpsons II surgical resection was achieved via a left curvilinear incision, with histology confirming WHO grade I meningioma. Figure 5 depicts intraoperative images of the cutting template, tumor resection, and implant fixation. No postoperative complications occurred.

Case 3

**Figure 6:**
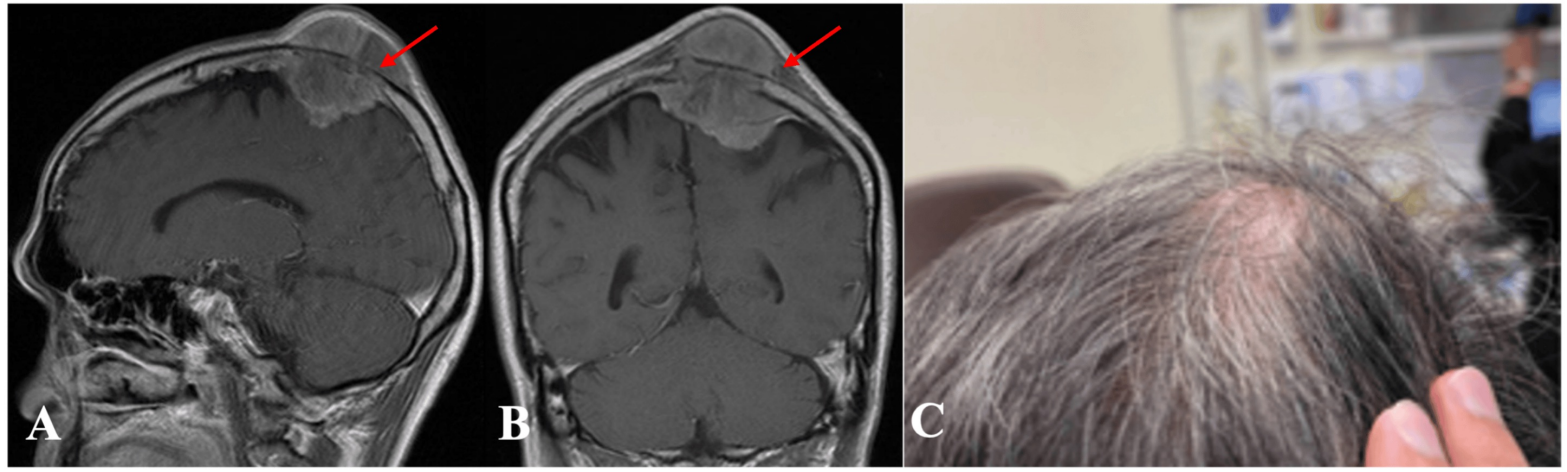
(A) Sagittal and (B) coronal preoperative images of MRI brain (T1-weighted with contrast) showing right parasagittal tumor (red arrow). (C) Clinical photo of cranial tumor MRI: magnetic resonance imaging

**Figure 7:**
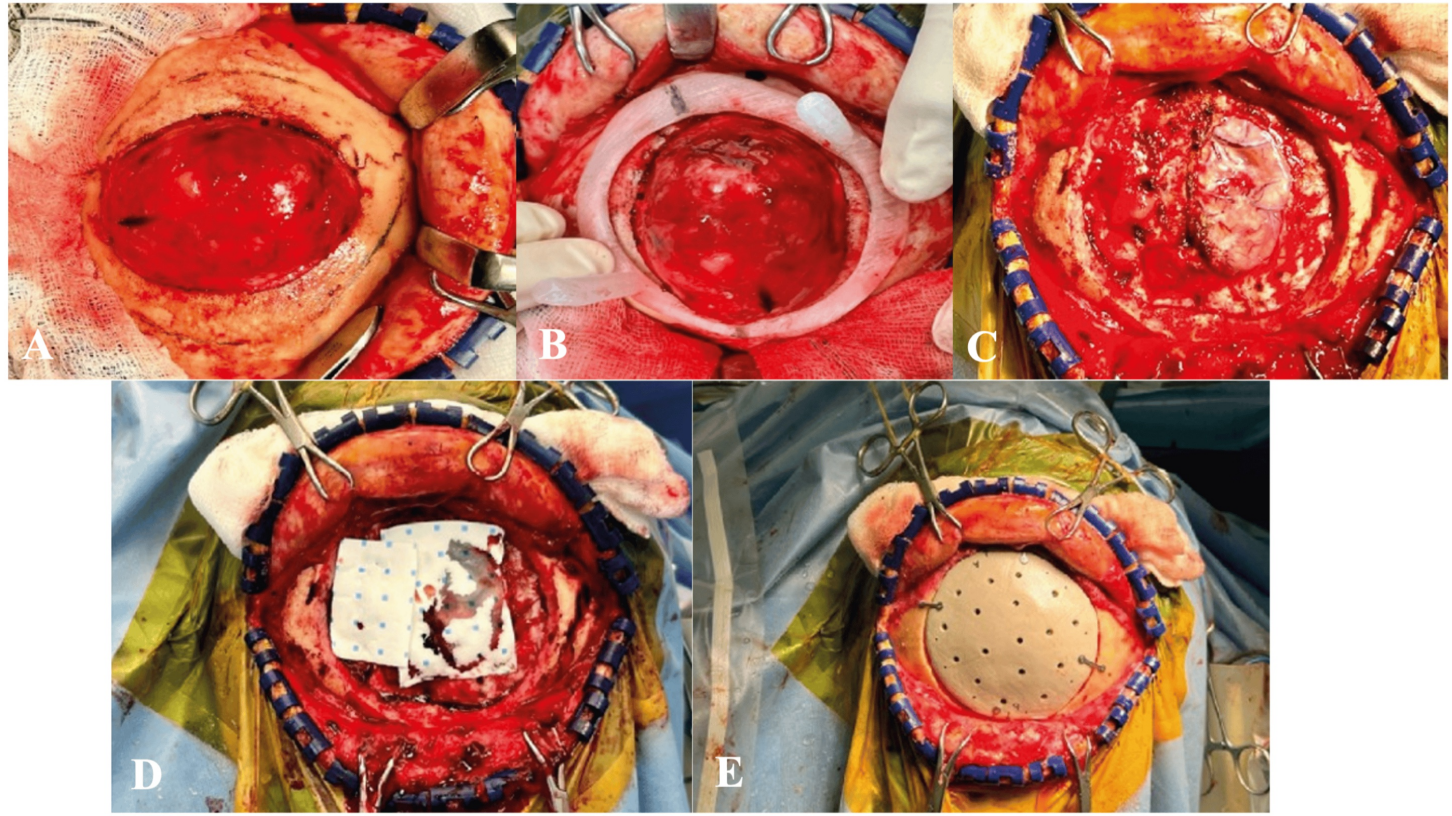
(A-E) Intraoperative images. (B) Cutting template positioned. (C, D) Post-craniectomy done with tumor resected followed by (E) PEEK implant fixation PEEK: polyetheretherketone

A 67-year-old man presented with an enlarging scalp lump over six months (Figure 6C). His medical history was significant for hypertension. MRI of the brain (Figures 6A, 6B) revealed a left convexity tumor with calvarial invasion: a left vertex mass measuring approximately 6.2 × 5.0 × 5.9 cm, involving the left parietal bone and an extra-axial component with dural tail. The lesion exerted local mass effect on the brain parenchyma and involved SSS. A Simpson II resection was performed through a bicoronal incision. Histopathological analysis confirmed a WHO grade 3 anaplastic meningioma. Figure 7 illustrates intraoperative images demonstrating the cutting template and the fixated PEEK implant. Postoperatively, an extradural collection formation was observed that required subsequent operative evacuation. Adjuvant radiotherapy, comprising 60 Gy delivered in 30 fractions to the surgical bed, was completed three months after resection, followed by GKS six months post-resection due to recurrence.

Case 4

**Figure 8:**
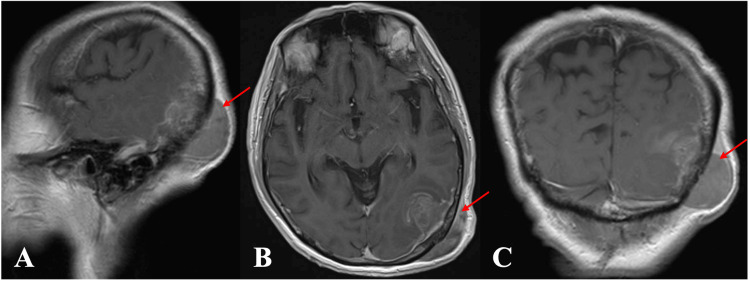
(A) Sagittal, (B) axial, and (C) coronal preoperative images of MRI brain (T1-weighted with contrast) showing right parasagittal tumor (red arrow) MRI: magnetic resonance imaging

**Figure 9:**
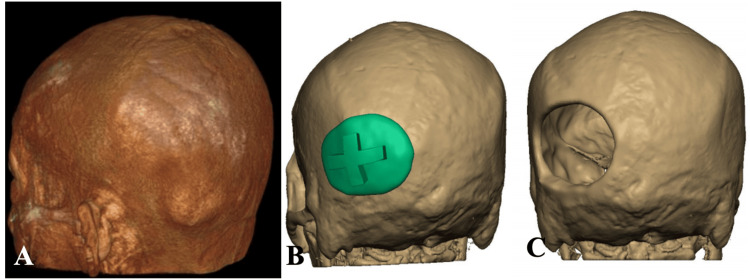
(A-C) Preoperative planning images showing calvarial lesion with image mapped out and cut out for generation of PEEK implant PEEK: polyetheretherketone

**Figure 10:**
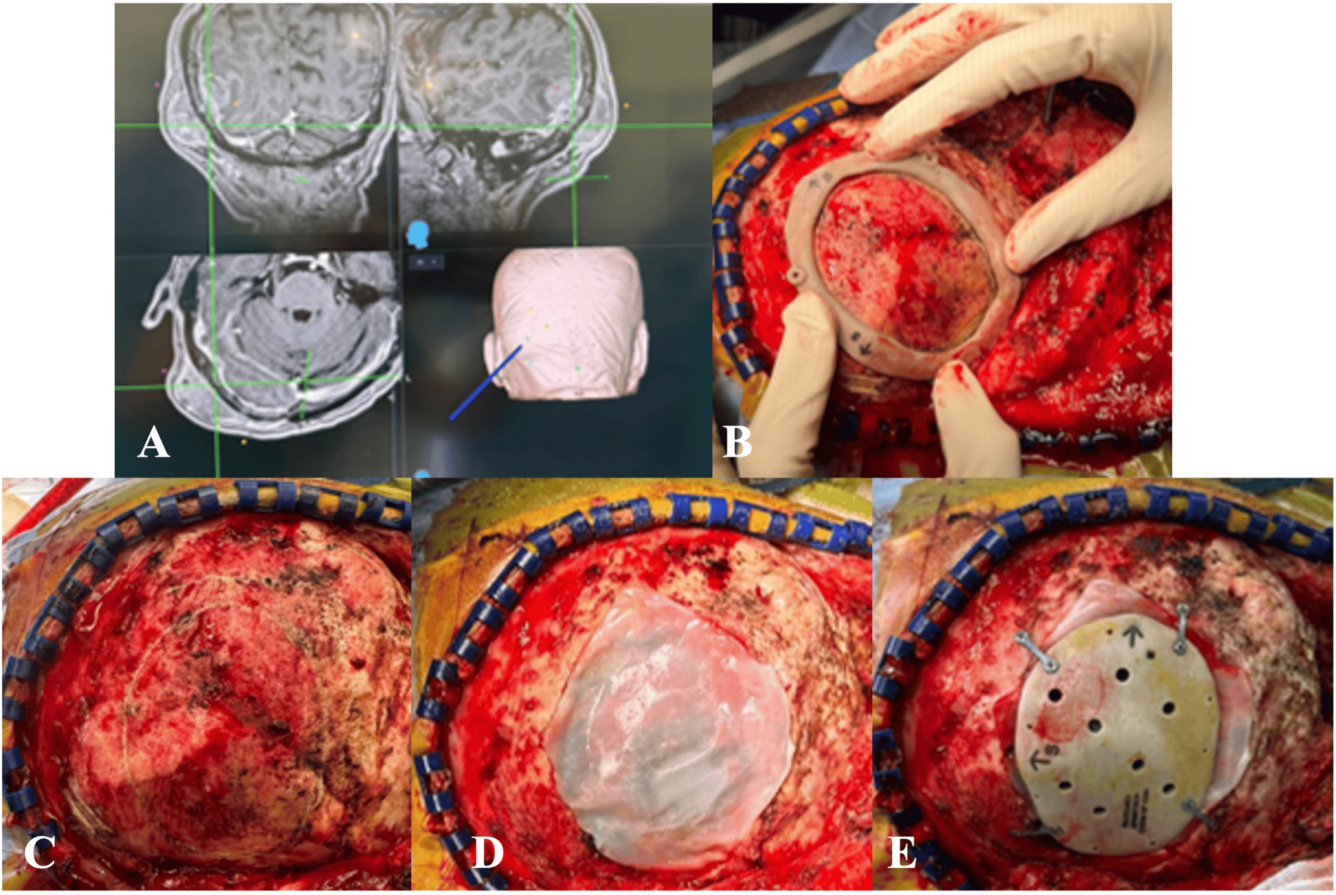
(A) StealthStation™ navigation images and surgical planning. (B, C) Cutting template position and marking. (D) Post-craniectomy with (E) PEEK implant fitted PEEK: polyetheretherketone

A 71-year-old man presented with a left occipital lump with a medical history of WHO grade 3 solitary fibrous tumor of the neck, previously excised as a cervical intramuscular mass. His comorbidities included coronary artery disease with stent insertion, hypertension, hyperlipidemia, gouty arthritis, thalassemia minor, and childhood asthma. MRI of the brain revealed a left occipital convexity tumor with calvarial invasion. The lesion measured approximately 2.8 cm and was associated with a small posterior dural lesion and abnormal signal intensity within the overlying parieto-occipital bones, contiguous with a large dominant subgaleal mass measuring approximately 4.1 cm (Figure 8). Figure 9 illustrates the planning images used for fabricating the PEEK implant. Histopathological examination revealed a high-grade malignant neoplasm consistent with a WHO grade III solitary fibrous tumor with a Ki67% proliferation index of more than 70%. Simpson grade II resection was performed through a hockey-stick incision. Figure 10A shows the intraoperative navigation images, while Figures 10B-10E illustrate the cutting template and fitting of the PEEK implant. The patient underwent palliative radiotherapy two months post-surgery, receiving volumetric modulated arc therapy delivering 36 Gy in 12 fractions to the recurrent left occipital tumor, and was subsequently started on oral tyrosine kinase inhibitors and chemotherapy. The patient died seven months post-surgery due to disease progression despite radiotherapy, immunotherapy, and chemotherapy.

Case 5

**Figure 11:**
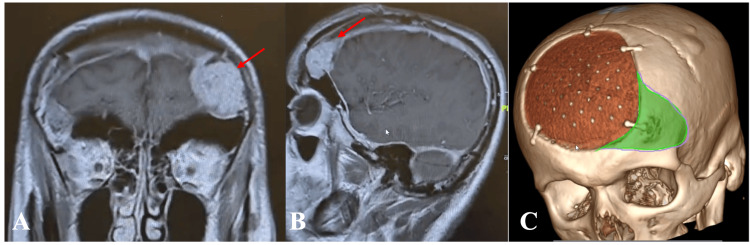
(A) Coronal and (B) sagittal preoperative images of MRI brain (T1-weighted with contrast) showing left frontal tumor (red arrow). (C) Preoperative planning images for the generation of template and PEEK implant MRI: magnetic resonance imaging; PEEK: polyetheretherketone

**Figure 12:**
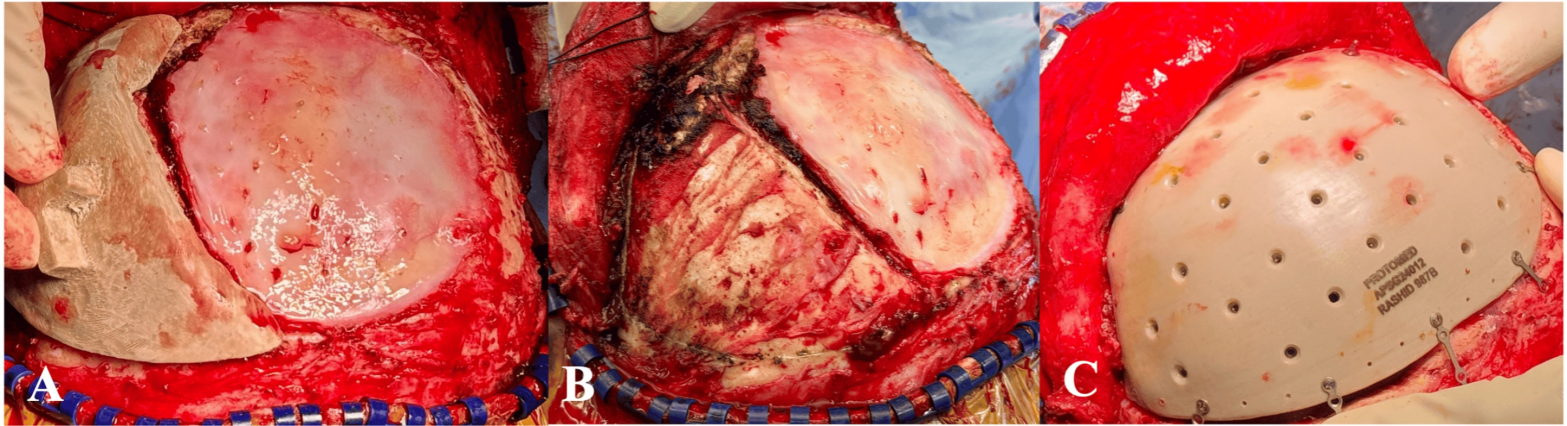
(A, B) Intraoperative images with cutting template and (C) PEEK implant fitted PEEK: polyetheretherketone

A 49-year-old man presented with headaches and had a medical history of recurrent left frontal convexity WHO grade II meningioma. The lesion recurred after a prior bifrontal craniectomy and resection of a bifrontal parasagittal meningioma, followed by cranioplasty in 2022 and ventriculoperitoneal shunt insertion in 2024. MRI of the brain (Figures 11A, 11B) revealed a left frontal convexity tumor with calvarial invasion-an avidly enhancing lesion expanding the left frontal bone and measuring approximately 2.7 cm. Thinning of the outer table and erosion of the inner table were observed, along with sheet-like dystrophic calcification of the underlying dura. Figure 11C shows the preoperative planning images for generating the PEEK implant. A Simpson grade I resection was performed through a bicoronal craniotomy, while histopathological examination confirmed a WHO grade II meningioma. Figure 12 illustrates the intraoperative images showing the fitted PEEK implant. Adjuvant radiotherapy was administered two months after resection.

A summary of each case showing the diagnosis, resection extent, Sloan classification, operative time, vendor used, intraoperative adjustments if any, complications, and further follow-up treatment is presented in Table 1. 

**Table 1:** Summary of each patient showing diagnosis, resection extent, Sloan classification, operative time, vendor used, intraoperative adjustments if any, complications, and further follow-up treatment GKS: Gamma Knife Surgery; RT: radiotherapy; TKI: tyrosine kinase inhibitor

Patient No.	Diagnosis	Resection extent	Sloan class	Operative time	Vendor	Intraoperative adjustments	Complications	Further treatment (1-year follow-up)
1	WHO grade I meningioma	Simpson II	2	4 hours 59 minutes	Synthes	No	Postoperative seizures	GKS 11 months post-surgery
2	WHO grade I meningioma	Simpson II	1	4 hours	Synthes	No	-	-
3	WHO grade III anaplastic meningioma	Simpson II	2	3 hours 8 minutes	Protomed	No	Postoperative extradural collection formation requiring operative evacuation	RT 3 months post-surgery, GKS 6 months post-surgery
4	WHO grade III solitary fibrous tumor	Simpson II	2	1 hour 8 minutes	Protomed	No	Demised 7 months post-surgery due to disease progression	Palliative RT 2 months post-surgery, TKI; pazopanib and chemotherapy 2 months post-surgery
5	WHO grade II meningioma	Simpson I	1	4 hours 52 minutes	Protomed	No	-	RT 2 months post-surgery

## Discussion

Advances in computer-aided design and planning systems, 3D printing technologies, and the availability of materials for customizable PSIs have broadened the scope of cranioplasty applications. The benefits of cranioplasty following stroke or trauma are widely established in neurosurgery, including maximized recovery potential, improved functional and cognitive outcomes, enhanced quality of life, and satisfactory cosmetic reconstruction [18,19]. The use of PSIs for reconstruction following osseo-invasive tumor resection has been documented in several case series of single-stage resection and reconstruction [20-22]. Additionally, studies report the advantages of using a cutting guide for planning craniectomy and optimizing cranioplasty fit during single-stage reconstruction following osseo-invasive tumor resection [23]. In this report, we present a commercially available PSI strategy applied to a case series from our institution.

Multiple materials are available for cranioplasty, with surgeon preference, material availability, and cost being factors influencing selection [24]. Hand-molded acrylic and titanium are commonly used for single-stage reconstruction following osseo-invasive tumor resection because they are readily available and cost-effective. However, titanium metal alloys exhibit a higher risk of implant exposure and failure, often necessitating reoperation [25-27]. While PMMA enables easy freehand modeling of implants, intraoperative hand modeling may result in cosmetic irregularities in large cranioplasties and significantly increase the operative time. Compared to bone cement, PMMA exhibits lower complication and infection rates [28]. Additionally, PMMA produces better postoperative imaging quality than titanium implants, which may obscure follow-up scans [28]. However, potential neurotoxic effects of PMMA during cranioplasty have been postulated due to the chemical toxicity of residual methacrylate monomer during the irrigation process, causing transient cranial neuropathies and delayed awakening with confusion from anesthesia [29]. Hydroxyapatite, a calcium phosphate-based material, is commonly used for its excellent integrative properties that promote osteointegration [30]. Although hydroxyapatite is associated with lower infection rates and fewer implant removals, its inherent fragility remains a major limitation and increases the risk of epidural hematoma formation [31-33].

Our institution has extensive experience using PEEK PSIs. PEEK is bioinert, mechanically durable, and produces minimal imaging artifacts, making it an ideal material for cranioplasty, particularly after tumor resection [10,34]. However, limitations include high cost, production time for PSI development, and the need for in-house sterilization. In osseo-invasive tumors-particularly malignant cases where surgery is time-sensitive-a two-stage cranioplasty procedure is typically preferred [7]. However, given that the turnaround time for manufacturing PEEK implants with our partner manufacturers is as short as one week, from imaging to in-house sterilization, the use of PEEK remains a reasonable option even in cases of suspected malignant tumors. A single-stage surgery is preferred over a staged approach to minimize incision reopening, promote faster wound healing, and enable quicker initiation of adjunctive systemic therapies [35].

Few case reports describe simultaneous cranioplasty following tumor resection of osseo-invasive tumors [20-22], typically involving a preplanned resection strategy and the use of intraoperative neuronavigation to guide craniectomy margins. However, this approach may still result in a suboptimal fit of the cranioplasty, necessitating additional handling and trimming of the implant intraoperatively to achieve an optimal fit.

In this context, incorporating a cutting guide based on the preoperative plan may help to optimize the craniectomy and improve the fit of the subsequent cranioplasty following tumor resection. Furthermore, the cutting guide includes orientation markers for intraoperative alignment of the final implant and prevents errors during craniectomy planning. Incorporating safety margins during initial planning facilitates intraoperative modification of the surgical approach and enables extension of the craniectomy, if necessary, within defined boundaries. Incorporating a resection template enhances intraoperative precision, reduces implant handling, and minimizes the risk of malfit. This was consistently observed in our series, with planned resections achieving high implant conformity without infection-related complications. In contrast, Lönnemark et al. report high infection and implant failure rates, with six of 36 patients developing implant-related infection requiring removal, and a mean time to infection of 220 days [36].

An optimal fit and satisfactory cosmesis remain key outcomes in cranioplasty. Patient-specific cranioplasty using a cutting template, demonstrated in our series of five patients, achieved excellent results, corresponding to Sloan Class 1 (no visible or palpable irregularity) or Class 2 (palpable but not visible irregularity not requiring reoperation) outcomes [17]. Improved cosmetic appearance following cranioplasty contributes to better self-esteem and enhanced social integration among postoperative patients [37]. Additionally, we present a case of reoperation demonstrating how the cranioplasty design was replanned and executed following tumor recurrence, as may occur in higher-grade recurrent lesions. Patient satisfaction regarding cosmetic outcomes is increasingly crucial, with quality-of-life assessments among postoperative patients emphasizing this aspect [38]. Future studies should incorporate these measures to quantify patient experience alongside clinical and cosmetic outcomes.

While the use of PSI with tailored resection templates has demonstrated positive outcomes, some limitations remain, including the small sample size of five patients. Furthermore, the absence of a control group limits the strength of comparative analysis. However, despite the small cohort, the combined use of a cutting template with PSI represents a promising approach in cranioplasty reconstruction.

## Conclusions

This report presents an institutional experience with commercially available PSIs and tailored resection templates for single-stage resection and reconstruction of osseo-invasive cranial tumors. Incorporating a cutting guide and resection template further enhances cosmetic outcomes by ensuring optimal implant fit while maintaining the feasibility of single-stage surgery. Additionally, PEEK exhibits multiple advantages as a cranioplasty material, making it the implant of choice at our institution compared to other available materials. The combination of PEEK cranioplasty with a cutting template demonstrated effectiveness in the five patients treated. Despite limitations of a lack of control group, as well as a small sample size with dependence on vendor turnaround time for timely implant production, this technique may offer a safe, precise, and efficient strategy for achieving optimal tumor control, functional recovery, and aesthetic outcomes, minimizing operative time and complications while enhancing patient satisfaction. Nonetheless, longer-term follow-up with a larger number of patients and further studies would provide stronger evidentiary support.
